# Inhibition of MMP14 potentiates the therapeutic effect of temozolomide and radiation in gliomas

**DOI:** 10.1002/cam4.104

**Published:** 2013-06-30

**Authors:** Ilya Ulasov, Bart Thaci, Purvaba Sarvaiya, Ruiyang Yi, Donna Guo, Brenda Auffinger, Peter Pytel, Lingjiao Zhang, Chung Kwon Kim, Anton Borovjagin, Mahua Dey, Yu Han, Anatoly Y Baryshnikov, Maciej S Lesniak

**Affiliations:** 1 The Brain Tumor Center, The University of ChicagoChicago, Illinois, 60637; 2 Department of Pathology, The University of ChicagoChicago, Illinois, 60637; 3 Department of Periodontology, School of Dentistry UABBirmingham, Alabama, 35294; 4 Institute of Experimental Biotherapy, NN Blokhin Research Cancer CenterRAMS Kashirskoe Shosse 24, Moscow, Russia

**Keywords:** Brain cancer, glioma, MMP14, radiation, temozolomide

## Abstract

Metalloproteinases are membrane-bound proteins that play a role in the cellular responses to antiglioma therapy. Previously, it has been shown that treatment of glioma cells with temozolomide (TMZ) and radiation (XRT) induces the expression of metalloproteinase 14 (MMP14). To investigate the role of MMP14 in gliomagenesis, we used several chemical inhibitors which affect MMP14 expression. Of all the inhibitors tested, we found that Marimastat not only inhibits the expression of MMP14 in U87 and U251 glioma cells, but also induces cell cycle arrest. To determine the relationship between MMP14 inhibition and alteration of the cell cycle, we used an RNAi technique. Genetic knockdown of MMP14 in U87 and U251 glioma cells induced G_2_/M arrest and decreased proliferation. Mechanistically, we show that TMZ and XRT regulated expression of MMP14 in clinical samples and in vitro models through downregulation of microRNA374. In vivo genetic knockdown of MMP14 significantly decreased tumor growth of glioma xenografts and improved survival of glioma-bearing mice. Moreover, the combination of MMP14 silencing with TMZ and XRT significantly improved the survival of glioma-bearing mice compared to a single modality treatment group. Therefore, we show that the inhibition of MMP14 sensitizes tumor cells to TMZ and XRT and could be used as a future strategy for antiglioma therapy.

Glioblastoma remains an incurable form of brain cancer. In this manuscript, we show that inhibition of MMP14 can potentiate the efficacy of current standard of care which includes chemo- and radiotherapy.

## Introduction

Glioblastoma multiforme (GBM), the most common primary brain tumor, is a devastating disease associated with high mortality 1. Although most of the patients partially respond to adjuvant chemotherapy such as TMZ and XRT, tumor recurrence is inevitable 2–3. Similar to other cancers, glioma initiation and its relapse result from aberrant activation of the genes responsible for tumor invasion, proliferation, and cell division, including MMPs 4–5. Membrane-bound MMPs such as MMP14 are induced in response to TMZ and XRT and are also associated with extracellular matrix degradation 6–7. Thus, understanding the regulation of MMP14 will help in the development of new approaches for anticancer therapy.

MMP14 (MT1-MMP) is a membrane-type metalloproteinase with collagenase activity and has been implicated to play a role in many biological processes in normal and tumor tissues 8 Although majority of the studies involving MMP14 primarily focus on angiogenesis 9–10 and invasion 11–12, recent studies have also pointed to the involvement of MMP14 in tumor proliferation 13 and remodeling of extracellular matrix and basement membrane 14.

In the present study, we explored the role of MMP14 in the pathogenesis of glioma. We show that MMP14 correlates with glioma aggressiveness and patient survival. Moreover, the MMP-14 inhibitor, Marimastat, induces glioma cell cycle arrest and slows tumor progression. Genetic silencing of MMP14 positively correlates with G2/M arrest and sensitizes glioma cells to TMZ and XRT in vitro and in vivo in an orthotopic glioma model.

## Material and Methods

### Patient specimen and ethical statement

To analyze the expression of MMP14 in glioma patient's tissues, we obtained a tissue microarray (TMA) provided by US Biomax (Rockville, MD) and supplemented these data by the staining of brain tumor specimens obtained from patients undergoing brain tumor resection at the University of Chicago Medical Center. These samples were collected from ongoing craniotomy, decoded and graded by neuropathologist. Each tissue specimen contained at least 90% cancer cells. The study was approved the Institutional Review Board (IRB) at the University of Chicago.

### Cell lines

The human gliomas U87, T98, and U118 were purchased from ATCC (Manassas, VA). GBM patient-based GBM39 and GBM43 were provided by Dr. D. James (University of California at San Francisco). The U251 cells were kindly provided by Dr. Atique Ahmed (The University of Chicago). N10 were obtained from Japan Tissue Bank (Tokyo, Japan). All cell lines were cultured in monolayer in T75 cm^2^ flasks in either MEM (for U87 and U118) or DMEM (U251, N10, T98) supplemented with 10% FBS (U87, U118, U251, N10, T98, and GBM39) or 1% (GBM43), 100 U/mL penicillin, 100 μg/mL streptomycin, at 37°C in a humidified atmosphere with 5% CO_2_.

### Drugs and treatments

CP101537, CP471474, and Marimastat were purchased from Sigma chemicals (St. Louis, MO) and dissolved either in dimethyl sulfoxide (DMSO) (CP101537 and CP471474) or in milliQ water (Marimastat) to obtain a stock concentration of 3.8 mMmol/L. The aliquots of the stock were stored in minus 20°C. TMZ was provided by Schering Plough-Merck (Whitehouse Station, NJ) and dissolved in DMSO to obtain a 100 mmol/L stock as suggested by the vendor. The radiation exposure was conducted by using the Gamma Cell 1000 (Nordion, Canada) unit. In vitro, cells were treated with 4 Gy daily for three consecutive days.

### Flow cytometry

Cell cycle analysis was done as previously described 15. Briefly, at selected time points, treated/untreated cells were fixed using 70% ethanol and then stored at minus 20°C for 24 h. The PI-stained nuclei cells (50 μg/mL of propidium iodide and 0.5 mg/mL of RNase A for 30 min at 37°C) were analyzed using a FACsaria (San Jose, CA). The distribution of the cells was evaluated by using the cell cycle template with (FloJo).

### Cell proliferation and cell viability assays

To determine the effect of drugs on glioma cell proliferation, plated cells were treated with DMSO, Marimastat, TMZ (100 μmol/L), XRT (4 Gy/daily, three consequent exposures), or a combination of them. At selected time points, cell proliferation was measured by 3-(4,5-dimethylthiazol-2-yl)-2,5-diphenyltetrazolium bromide (MTT) assay (Cell proliferation kit I, Roche, Indianapolis, IN) by adding 20 μL of MTT substrate to each well. After 3 h of incubation at 37°C and 5% CO_2_, 100 μL of sodiumdodecyl sulphate (SDS)-based stop solution was added to each well to stop the enzymatic reaction. On the following day, the absorbance was measured by the Multiscan MS microplate reader with a reference at 650 serving as a blank.

### Lentivirus design and infection

Target cells were plated 24 h before infection and next day transduced with either shRNA plasmids (shScramble: GCACTACCAGAGCTAACTCAGATAGTACT; MMP14sh2: CTACCACAAGGACTTTGCCTCTGAAGGCC; MMP14sh4: ATGGAAATGACATCTTCCTGGTGGCTGTG; MMP14sh5: ACTCTGCCGAGCCTTGGACTGTCAGGAAT (Origene USA, Rockville, MA) or infected with mission shRNA lentivirus transduction particles containing either control shRNA (SHC002V, CAACAAGATGAAGAGCAC CAACTCGAGTTGGTGCTCTTCATCTTGTTGTTTT) or shRNA specific for human MMP14 gene (NM_004995.2,CCGGCGGCCTTCTGTTCCTGATAAACTCGAGTTTATCAGGAACAGAAGGCCGTTTTTG, Sigma Chemicals) for 48 h at 37°C and 5% CO_2_ in the presence of 8 μg/mL of hexadimethrine bromide (Sigma). Efficacy of the knockdown was checked by Western.

### miRNA isolation and quantitative PCR

Total miRNA was isolated by using miRNeasy isolation kit (Qiagen, Dusseldorf, Germany) and then transcribed by using qScript microRNA cDNA synthesis kit (Quanta Biosciences, Gaithersburg, MD). To determine the expression of miRNAs induced by TMZ and XRT, we utilized a miFinder SAbioscience array. The fold expression of each microRNAs was evaluated based on normalization 16. The expression of selected miRNAs was validated via real time polymerase chain reaction (PCR) using Opticon 2 Instrument (Bio-Rad, Hercules, CA), SYBRgreen SuperMix (Quanta Biosciences), gene-specific primers synthesized by (www.RealTimePrimers.com) and microRNA PCR assays (Quanta Biosciences). Relative expression of target genes was normalized to the expression of RNU6 house keeping miRNA. Each sample was analyzed in triplicates. The analysis of experimental data was done using ΔΔCt method.

### Western blotting

To detect expression of MMP14, cytosolic fraction (supernatant prepared using MEM-PER membrane protein extraction kit, Thermo Scientific, Hanover Park, IL) was mixed with 4× Laemmle buffer (Boston Bio product, MA) and subjected to 4–20% SDS-PAGE (sodiumdodecyl sulphate polyacrylamide gel electrophoresis) precast gel (Bio-Rad, Hercules, CA). Forty micrograms of the extracted proteins were transferred to the polyvinylidene frouride (PVDF) membrane and were probed with anti-MMP14 antibody (Abgent, San Diego, CA). In addition, blots were probed with antiactin antibody (AC-15, Sigma) for detection of β-actin as a loading control. Membrane-bound primary antibodies were detected by either horseradish peroxidase-conjugated secondary anti-mouse (sc-2031, Santa Cruz Biotechnology, Santa Cruz, CA) or anti-rabbit (sc-2030, Santa Cruz Biotechnology) antibodies. The protein bands were detected using Western C enhanced solution (Bio-Rad, Hercules, CA) and images were acquired by using gel doc documentations system (Bio-Rad).

### In vivo model

Six-week-old female athymic nude mice (Charles River, Wilmington, MA) were used in these studies. To evaluate the impact of MMP14 knockdown on glioma growth in vivo, we established brain tumor xenografts by using 2.5 × 10^4^ cells (U87shScramble, U87shMMP14, U251shScramble, and U251shMMP14) per mouse. To determine the survival of glioma bearing mice in the presence of TMZ and XRT, 2.5 × 10^4^ U251shScramble or U251shMMP14 cells were implanted intracranially. Seven days later, each group of the mice was randomly divided into two groups to receive treatment with either DMSO or combination of TMZ (5 mg/kg, i.p.) followed by XRT, 2 Gy/kg with a 3 h lag time between treatments as suggested by Galban et al. 17. All treatments were administered once a week for a 5-week period. Based on our preliminary dose-escalating experiments, we identified that TMZ (5 mg/kg) and 2 Gy/kg as minimally efficacious doses at multiple doses instead of one weekly dose and the schedule combination was chosen for our GBM mouse model to yield enhanced survival in combination with MMP14 attenuation. The survival rate of the mice was determined at the end of the experiments and the results were plotted as Kaplan–Meier survival for each of the group.

### Immunohistochemistry

Immunoassaying was conducted on formalin-fixed paraffin embedded tissue specimens. After standard antigen retrieval, slides were incubated with either Ki-67 (Thermo Scientific) or anti-MMP14 antibodies (Abgent) following by incubation with VECTASTAIN Elite ABC Kit (Vector laboratories, Burlingame, CA). To acquire IHC images and calculate tumor volume, slides were scanned using 3D Histech Panoramic Scanner (Perkin Elmer, Downers Grove, IL) followed by analyses with Panoramic view software as proposed by Grommes et al. 18. To evaluate the expression of MMP14 in the clinical settings we used a three tier score. We defined our scoring based on percentage of the cells expressing MMP14: “0,” <5% cells; “1+,” 5–20%; “2+” 20–40%; “3+,” >40%.

### Statistical analysis

All experiments have been performed at least twice in triplicates under identical conditions. Data are expressed as mean ± SEM. All statistical tests were performed using GraphPad Prism 4 or STATA 12 (GraphPad Software, Inc., La Jolla, CA). A *P-*value less than 0.05 was considered statistically significant.

## Results

### MMP14 expression is significantly increased in TMZ/XRT-treated glioma tissues

**Figure 1:**
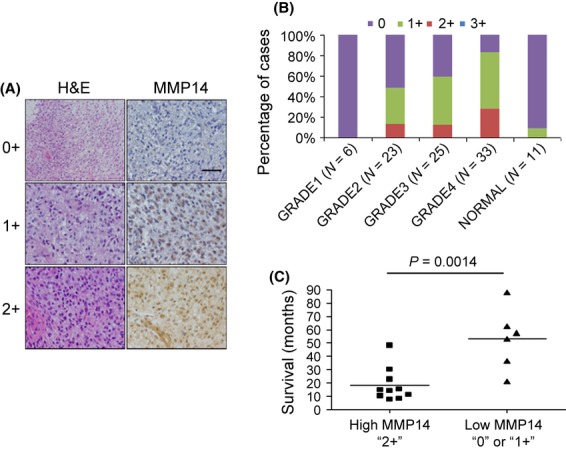
Upregulation of MMP14 prolongs survival of glioma patients. (A) Relationship between MMP14 expression and glioma progression was evaluated using hematoxylin and eosin (H&E) and MMP14 staining of glioblastoma multiforme (GBM) specimens obtained at the University of Chicago. Magnification 400×; (B) Distribution of MMP14 expression in different grades of brain tumors (*N* = 98 in total); (C) MMP14 affects mean survival time of GBM patients. Eighteen clinical samples were stained with MMP14, scored using three tier system and classified into low MMP14 (IHC score “0” and “1+”) or high MMP14 group (score “2+” and “3+”); Fisher's exact test (*P < 0.05, **P < 0.01, ***P < 0.001).

Accumulating evidence suggests that exposure of the tumor cells to chemotherapeutic drugs causes upregulation of stress response genes such as proinflammatory cytokines and MMPs 19–20. As the treatment of brain tumor cells with chemotherapy has been shown to induce the expression of MMPs 21, we wanted to determine if there was a link between the expression of MMPs and the mortality of the patients with brain tumors. To establish the correlation between MMP14 expression and glioma grade, we stained a TMA containing 98 glioma samples with MMP14 antibodies in duplicates. The representative images and distribution of the MMP14-positive cases among clinical samples were plotted (Fig. 1A and B). Specifically, the percentage of MMP14-positive cases having “1+ and 2+” scores increased from 43% in grade 2 (*n* = 23, *P* < 0.001 vs. grade4) to 60% in grade 3, (*n* = 25, *P* < 0.05) and 80% in grade 4 (*n* = 33, *P* < 0.05).

Despite a clear correlation between MMP14 expression and tumor grade, it remains unclear whether expression of MMP14 has any prognostic significance. To determine that, we stained a set of surgically resected GBM specimens obtained at the University of Chicago. We separated the GBM samples based on low (“0” and”1+”) and high (“2+”) expression of MMP14 and calculated the average of monthly survival based on medical records. As shown in Figure 1C, patients with high MMP14 immunoreactivities had shorter mean survival time compared to those with low MMP14 immunoreactivities (18.25 ± 3.98, *n* = 10 vs. 53.22 ± 9.39, *n* = 7, *P* = 0.0014). Data presented in Figure 1B and C accounts for all GBM specimens, however, does not take into consideration the impact of TMZ/XRT therapy on glioma progression and expression of MMP14. Therefore, we divided 18 GBM specimens into TMZ/XRT treated and untreated group and evaluated distribution of MMP14 stainings. As seen in Figure S1, patients with treated glioblastoma exhibit strong level of MMP14 immunoreactivity compared to the untreated cases.

### Marimastat inhibits the proliferation of glioma cells

**Figure 2:**
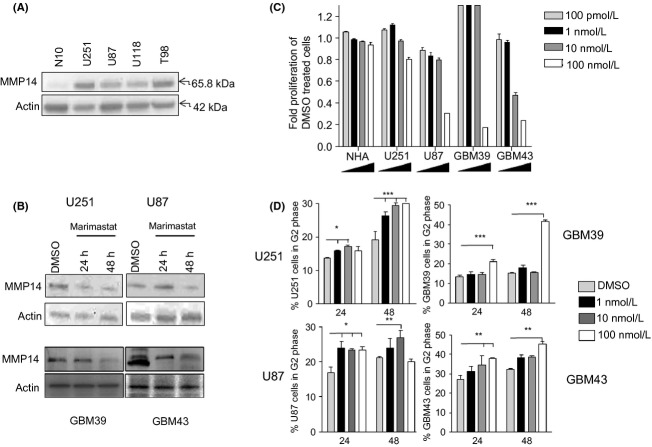
Marimastat downregulates MMP14 expression and induces G_2_/M cell cycle arrest in gliomas. (A) and (B) Western blotting analyses of MMP14 expression in glioma cells (N10, U87, U251, U118, T98, GBM39, and GBM43) or presence of 100 nmol/L of Marimastat in U251, U87, GBM39, and GBM43 tumor cells. Actin serves as a loading control; (C) The rate of glioma proliferation was measured after treatment with different doses of Marimastat; (D) Percentage of Marimastat-treated glioma cells located in G_2_ phase of cell division. Two-way analyses of variance (ANOVA) followed by Bonferonni post hoc test (*P < 0.05, **P < 0.01, ***P < 0.001).

As the studies mentioned above suggest that MMP14 is upregulated during the course of therapy, we wanted to demonstrate whether MMP14 targeting can be a feasible antiglioma approach. To evaluate that, we treated glioma cells with a panel of MMP14 inhibitors CP101537, CP471474, and Marimastat, (Sigma-Aldrich Corp) that have previously been tested in treatment of glioma 22,23. We selected U87, U251, and patient-derived GBM39 and GBM43 cells because of the difference they exhibit in the expression level of MMP14 (Fig. 2A) and sensitivities to the treatment with TMZ and XRT 25–26. Among the drugs (Marimastat, CP101537, and CP471474) that were preliminarily tested on U87 and in U251 cells, only Marimastat demonstrated inhibition of MMP14 expression (data not shown). As seen in Figure 2B, the treatment of gliomas with 100 nmol/L of Marimastat significantly inhibits the expression of MMP14 in U251, U87, GBM39, and GBM43 tumor cells. To evaluate whether the inhibition of MMP14 affects cell proliferation, we performed an MTT assay (Fig. 2C). In this experiment, we observed that GBM39, GBM43, and U87 cells have higher sensitivity to Marimastat compared to the U251 cells. Interestingly, Marimastat specifically inhibited the growth of glioma cells and had no effect on normal human astrocytes (NHA). Recently, it has been suggested that MMPs are involved in cell divisions 27. To understand whether inhibition of glioma proliferation with Marimastat will affect the cell cycle, we stained Marimastat-treated glioma cells with PI dye. We found that Marimastat alters cell division of U87, U251, GBM39, and GBM43 glioma cells by increasing the number of tumor cells in G_2_ phase (Fig. 2D). Although the effect was most prominent in the presence of 100 nmol Marimastat, treatment of glioma cells with 1 and 10 nmol of Marimastat demonstrated G_2_ arrest as well.

### Effects of MMP14 genetic inhibition on cell division

**Figure 3:**
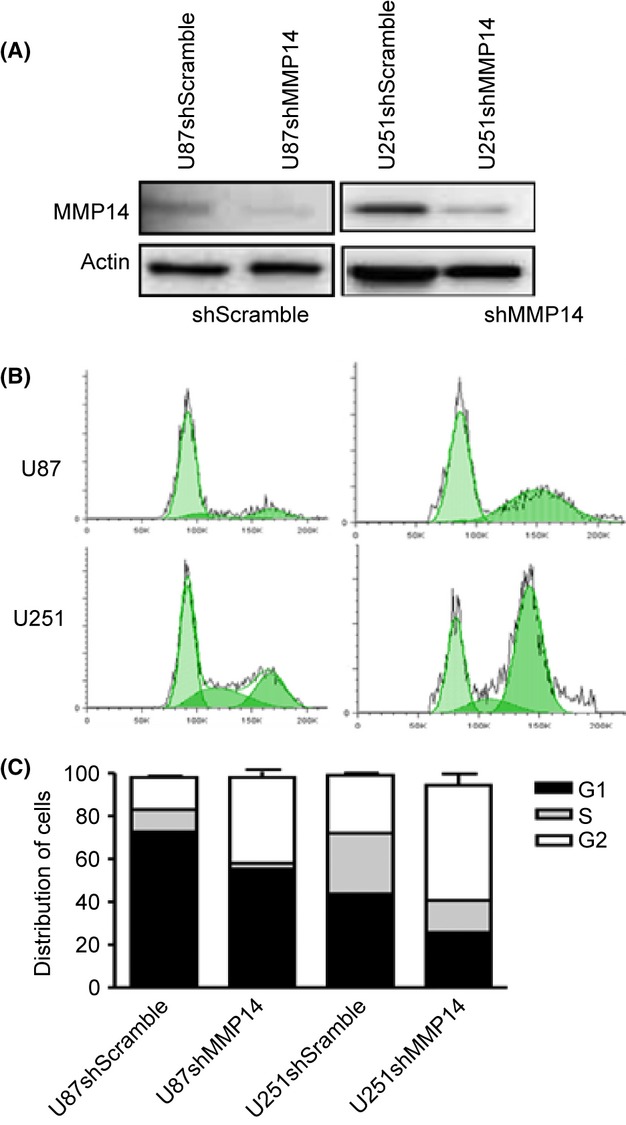
Genetic silencing of MMP14 results in glioma G_2_/M arrest. (A) Glioma cells were stably transduced with either shScramble or MMP14 shRNAs and were examined for the expression of MMP14. Actin serves as loading control; (B) and (C) Flow cytometry analyses of cell distribution in the presence of attenuated form of MMP14. Student *t-*test with Bonferroni correction (*P < 0.05, **P < 0.01, ***P < 0.001).

To investigate whether MMP14 regulates cell division, we knocked down U87 and U251 glioma cells using shRNA technology. We observed that protein and mRNA levels of MMP14 were significantly decreased in the presence of anti-MMP14 shRNAs (Fig. S2). Moreover, silencing of MMP14 inhibited expression of downstream targets such as MMP2. To establish a relationship between inhibition of MMP14 and cell cycle arrest, we rescued a panel of stable MMP14 knockdown U87 and U251-based cells along with scrambled transfected clones (Fig. 3A). Inhibition of MMP14 with shRNA silencing led to cell cycle arrest of glioma cells in G_2_ phase (Fig. 3B and C). The number of cells in G_2_ phase increased from 15.1 ± 0.7 to 40.6 ± 3.9 (*P* < 0.001) for U87, and from 27 ± 0.8 to 53.83 ± 5.2 (*P* < 0.01) for U251 glioma cells.

### TMZ and ionizing XRT regulate expression of MMP14 via miRNA374B

**Figure 4:**
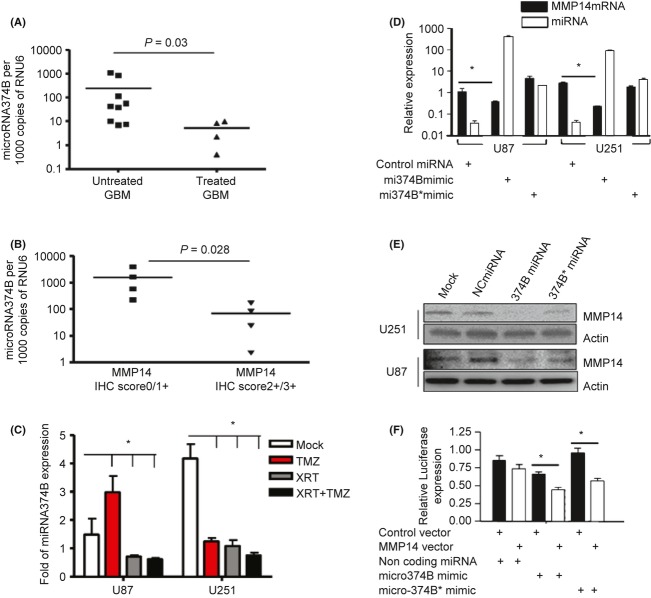
Temozolomide and radiation regulate MMP14 expression via microRNA374B in vitro and in vivo. Real time PCR analyses of miRNA374B expression in clinical samples (A) and in relation to MMP14 expression (B); In vitro, temozolomide and radiation treatment downregulate miRNA374B (C); Transfection of U87 and U251 reduces MMP14 expression at mRNA (D) and protein (E) levels; Direct binding of miRNA374B to 3′UTR of human MMP14 decreases luciferase expression at U251 (F).

To identify profile of glioma-based miRNAs which regulate expression of MMP14 and are altered by TMZ/XRT treatment, we applied the miFinder SAbioscience platform to the total miRNAs isolated from U87 cells treated with TMZ/ XRT (100 μmol/L+2 Gy/daily) or DMSO for 48 h. Based on relative quantification method (mean fold change >2 or <2), bioinformatical analyses, we determined that miRNA374B (−3.38-fold), miRNA374A (−2.1-fold), and miRNA150 (−4.1-fold) were decreased in treated U87 cells versus control groups. To validate that we measured expression of selected miRNAs using untreated/treated primary glioma specimens. As is shown in Figure 4A and B, miRNA374B expression correlates with a treatment status of patients and inversely with expression of MMP14. In vitro, treatment of U87 and U251 cells decreased expression of miRNA374B (Fig. 4C). Moreover, transfection of U87 and U251 with miRNA374B mimic downregulated MMP14 (Fig. 4D and E). Furthermore, binding of miRNA374B mimic to the 3UTR-MMP14 significantly reduced expression of luciferase in U251 cells, suggesting a strong relationship between MMP14mRNA expression and miRNA374B (Fig. 4F).

### The suppression of MMP14 in combination with TMZ and XRT improves the antiglioma effect in vitro and in vivo

**Figure 5:**
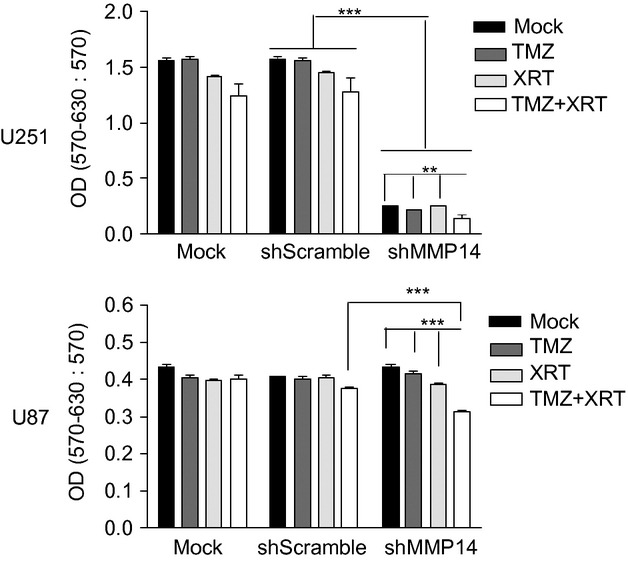
Temozolomide (TMZ) and ionizing radiation (XRT) cooperate with inhibition of MMP14. U87 and U251 cells treated with MMP14 shRNA and combination of radiation with temozolomide treatment exhibit a reduction in cell viability. Scramble or MMP14 stable knockdown glioma cells were treated with TMZ (100 μmol/L), XRT, or both therapies for 96 h. Two-way ANOVA followed by Bonferroni post hoc test (**P < 0.01, ***P < 0.001).

As previously shown in Figure 1, MMP14 is expressed more in TMZ/XRT treated rather than untreated GBMs. To assess the impact of MMP14 knockdown as an anticancer approach, we exposed U87 and U251 MMP14 knockdown cells to TMZ and XRT, and evaluated the effect of TMZ and XRT on cell viability by MTT assay. Compared to the cells transduced with scrambled shRNA, we observed that silencing of MMP14 inhibited the proliferation of tumor cells when treated with TMZ or XRT alone, as well as in combination (Fig. 5). These data demonstrate that MMP14 downregulation causes cell cycle arrest, and thus enhances the antiproliferative effect of chemotherapeutic agents such as TMZ and XRT.

**Figure 6:**
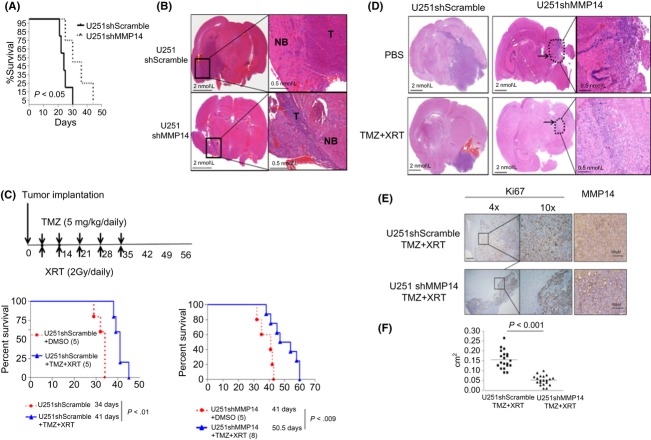
MMP14 knockdown enhances antiglioma effect mediated by temozolomide (TMZ) and ionizing XRT in U251 glioma model. (A) and (C) Cytotoxic effect of MMP14 silencing on human glioblastoma cells in vivo. The survival of U251 MMP14 knockdown or U251sh Scramble intracranial tumor-bearing mice was monitored for 55 days after injection; C) Nude mice received intracranial implantation of either U251shScramble or U251shMMP14 stable expressing glioma cells. Seven days after intracranial implantation of either U251shScramble or U251shMMP14 stable cells, mice were divided for control treatment (DMSO) or combination of 5 rounds of TMZ (5 mg/kg/daily+2 Gy/daily). Mice were monitored for next 55 days after tumor implantation and Kaplan–Meier survival curve was performed to determine the impact of MMP14 silencing on contribution to the TMZ/XRT therapies. Log-rank test: *P < 0.05, ** *P* < 0.01; (B) and (D) hematoxylin and eosin (H&E) staining was performed on paraffin sections of mice brains with established U251shScramble and U251shMMP14 or treated with TMZ/XRT therapy relatively; (E) and (D) Analysis of Ki-67 and MMP14 expression in the tumor sections of animals treated with TMZ/XRT and tumor volume in square centimeters is shown using two animals per group (10 sections/HE stain per block).

It has been recently reported that MMP14 is upregulated in response to TMZ and XRT exposure 21 and silencing of MMP, including MMP14, decreases glioma tumor formation in vivo 28–29. To evaluate the tumorigenic activity of MMP14 ablated U87 and U251 cells, we implanted either scrambled or MMP14 attenuated cells intracranially (Figs. 6A and S3). We observed that in both U87 and U251 cell lines, silencing of MMP14 prolonged survival of mice with brain tumor xenografts (U87, *P* < 0.01; U251, *P* < 0.05). Moreover, silencing of MMP14 decreased formation and invasion of U251 cells in the brain (Fig. 6B). To assess whether TMZ and XRT will benefit from MMP14 silencing, we divided U251 glioma-bearing mice into four different treatment groups: shScramble, shMMP14, shScramble+TMZ+XRT, and shMMP14+TMZ+XRT. These mice were then treated with the indicated drugs and their survival was monitored. As demonstrated in Figure 6C, treatment of mice carrying U251shScramble xenografts with TMZ+XRT improved survival compared to the DMSO-treated animals (*P* < 0.01). Additionally, we demonstrated that treatment of the mice with U251shMMP14 xenografts with TMZ+XRT improved survival compared to the DMSO-treated animals implanted with U251shMMP14 cells (*P* < 0.05). Finally, silencing of MMP14 in U251 model significantly prolonged survival of mice treated with TMZ+XRT compared to the U251 shScramble (*P* < 0.009) which results in low glioma proliferation (Fig. 6D, E, and F).

## Discussion

Glioblastoma multiforme has a poor prognosis due to low sensitivity of tumor cells to the current therapeutic options such as TMZ and XRT 30. One way to improve application of therapeutic modalities would be to identify genes responsible for brain tumor relapse and progression, and designing potential therapeutic approaches to inhibit their activity. Although the mechanism of tumor recurrence is still under investigation, it has been noticed that MMPs play an important role in tumor proliferation and invasion in many cancers including gliomas 31,32. Recently, screening for MMP in clinical samples identified that some of these including MMP-2 and 9 are upregulated in tumor tissue compared to normal tissue 34,35 and their expression predicts tumor recurrence in several types of cancers 37,38. Moreover, some reports recently suggested that expression of MMP14 downstream target such as MMP2 correlates with cancer progression in colon and breast cancers 11–40. In brain tumors, MMP2 serves as a predictor of tumor recurrence 32. However, the interpretation of data may be very challenging as they do not account for the impact of conventional therapy such as TMZ and XRT. To demonstrate that MMP14 staining exhibits strong correlation with glioma progression, we performed an IHC staining of 98 specimens with MMP14 antibodies. The results are consistent with the data published earlier 21 and imply that both of these therapeutic modalities increase the level of MMP14 which correlates with survival of glioma patients. Hence, reducing MMP14 may improve the effects of therapeutic modalities.

To understand how the inhibition of MMP14 affects TMZ and XRT, we knocked down the expression of MMP14 in several glioma cell lines. As expected, the treatment of glioma cells induced G_2_/M arrest at 24–48 h. To determine the relationship between MMP14 and cell cycle, we knocked down the MMP14 gene and performed cell cycle analysis via flow cytometry. Consistent with Marimastat inhibition data, silencing of MMP14 also induced G_2_/M arrest in all three glioma cell lines used in the study. Since U87 and U251 cells were established from various glioma patients having different mutations and growth properties, they may represent different subclasses of glioma. Hence, our results may reveal a general phenomenon for various glioma subtypes.

Gliomagenesis is a complex process that relies on the intricate interplay between uncontrolled cell proliferation and microenvironmental factors, such as provision of growth factors and matrix reorganization. In all these steps, MMPs play a crucial role and therefore their inhibition can delay tumor formation or its experimental counterpart: xenografts establishment in animal models. In our study, we show that MMP14 shRNA interference delays tumor formation in the U251 cells and completely inhibits glioma establishment in the U87 model. Moreover, treatment of U251ko xenografts with TMZ and XRT extended their survival further, proving that such therapies can be given in combination for increased benefit. The different sensitivity of MMP-14 interference in tumor formation is to be expected considering glioma cells express a wide range of MMPs and MMP-14 contribution could vary between glioblastoma subtypes 41. Henceforth, it is important to study the effect of MMP inhibition within different subtypes, once such models become available. That would help design individualized clinical trials to tease out the full effects of MMP inhibition since it has been shown that combination therapy of MMP-14 inhibitor with chemotherapy prolonged survival of GBM patients while Marimastat alone had no benefit in recurrent GBM 42.

In conclusion, we demonstrate that the attenuation of MMP14 inhibits glioma proliferation and improves therapeutic effect mediated by TMZ and XRT. Given the fact that some potential anticancer drugs which are in preclinical evaluation induce G_2_/M arrest, combining them with MMP14 inhibition could provide a rationale to improve efficacy of their application. Thus, the inhibition of MMP14 using chemical inhibition or via genetic silencing is an alternative approach for antiglioma therapy.

## Supporting Information

**Figure S1.** Distribution of MMP14 protein expression in untreated and TMZ/XRT treated GBM samples. Ten primary GBM and 8 recurrent GBM cases have been stained with antibodies recognize MMP14 human antigen. *P* = 0.055, Fisher's exact test. **Figure S2.** MMP14 downregulation induces G2M arrest. (A) U251 and U87 cells were transfected with different siRNAs targeting MMP14. Quantitative RT-PCR was carried out to verify the suppression of MMP14. (B) U251 and U87 were transiently transfected with either non-targeting shRNA (shScramble) or MMP14 sh4 (MMP14sh4). Western blotting was carried out to verify the suppression of MMP14. **Figure S3.** MMP14 shRNA increases survival of U87 glioma bearing mice. Log rank test *P* < 0.001.
