# Supplementary figures and images for: Inhibition of MMP14 potentiates the therapeutic effect of temozolomide and radiation in gliomas
Source: Cancer Med. 2013 Jun 30;2(4):457–67. doi: 10.1002/cam4.104 (PMC3799280; doi:10.1002/cam4.104)

Supplemental Figure 1


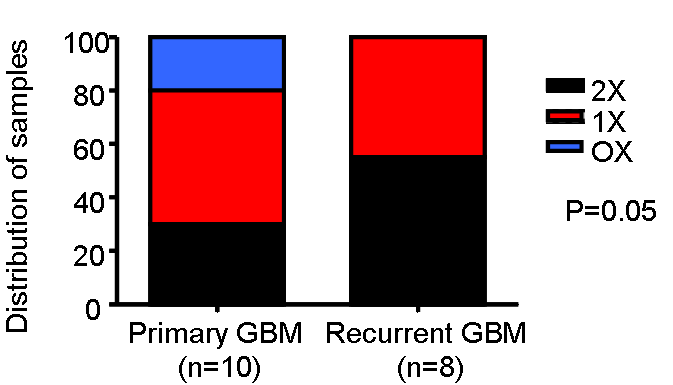


Supplemental Figure 2


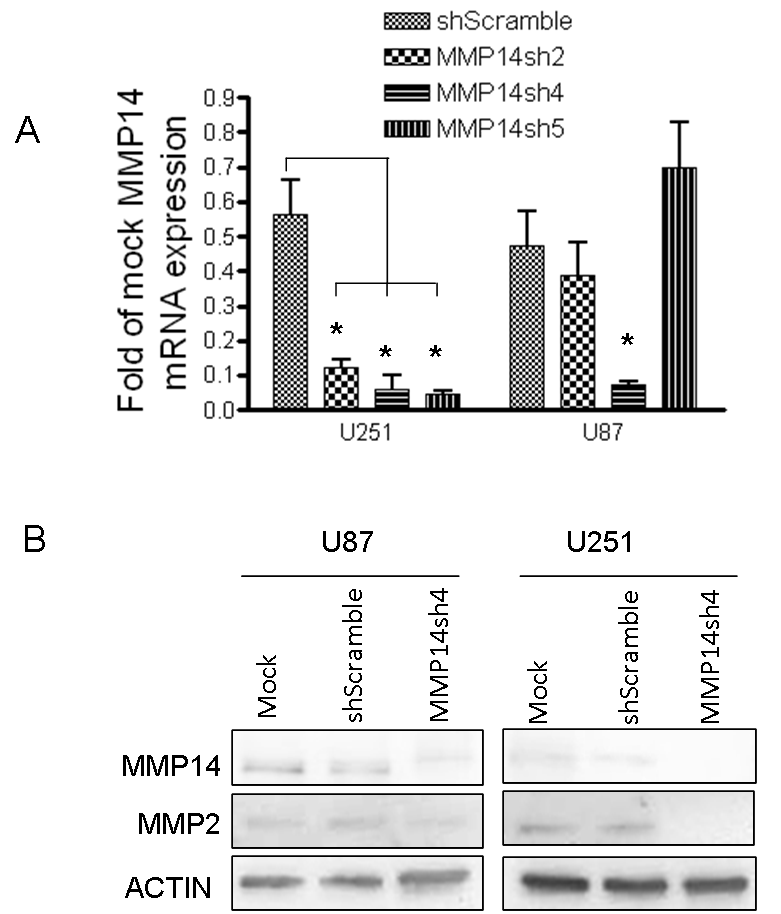


Supplemental Figure 3


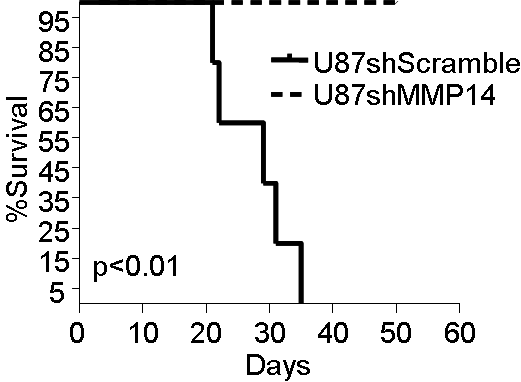

Supplement: Supplementary file 1 — Figure S1. Distribution of MMP14 protein expression in untreated and TMZ/XRT treated GBM samples. Ten primary GBM and 8 recurrent GBM cases have been stained with antibodies recognize MMP14 human antigen. P = 0.055, Fisher's exact test. Figure S2. MMP14 downregulation induces G2M arrest. (A) U251 and U87 cells were transfected with different siRNAs targeting MMP14. Quantitative RT-PCR was carried out to verify the suppression of MMP14. (B) U251 and U87 were transiently transfected with either non-targeting shRNA (shScramble) or MMP14 sh4 (MMP14sh4). Western blotting was carried out to verify the suppression of MMP14. Figure S3. MMP14 shRNA increases survival of U87 glioma bearing mice. Log rank test P < 0.001. [file cam40002-0457-SD1.docx]
