# Supplementary material for: Inhibition of MMP14 potentiates the therapeutic effect of temozolomide and radiation in gliomas
Source: Cancer Med. 2013 Jun 30;2(4):457–67. doi: 10.1002/cam4.104 (PMC3799280; doi:10.1002/cam4.104)
Supplement: Supplementary file 2 [file cam40002-0457-SD2.doc]

**Supplemental Figure**

**S1. Distribution of MMP14 protein expression in untreated and TMZ/XRT treated GBM samples**. Ten primary GBM and 8 recurrent GBM cases have been stained with antibodies recognize MMP14 human antigen. p=0.055, Fisher’s exact test.

**S2. MMP14 down-regulation induces G2M arrest.** (**A**) U251 and U87 cells were transfected with different siRNAs targeting MMP14. Quantitative RT-PCR was carried out to verify the suppression of MMP14. (**B**) U251 and U87 were transiently transfected with either non-targeting shRNA (shScramble) or MMP14 sh4 (MMP14sh4). Western blotting was carried out to verify the suppression of MMP14.

**S3. MMP14 shRNA increases survival of U87 glioma bearing mice.** Log Rank test p<0.001
